# The correlation between airborne pollen and asthma in children: a systematic review and meta-analysis

**DOI:** 10.3389/fped.2025.1614071

**Published:** 2025-06-23

**Authors:** Xiaoya Wang, Bo Liu, Yujie Yang, Jimin Li, Ziyan Tian, Jinwei He, Yuanxia Li

**Affiliations:** ^1^ Yan’an Medical School of Yan’an University, Yan’an, Shaanxi, China; ^2^ Department of Pediatrics, Yanan University Affiliated Hospital, Yan'an, Shaanxi, China

**Keywords:** airborne pollen, asthma, children, meta-analysis, systematic review

## Abstract

**Background:**

Childhood asthma is a prevalent chronic respiratory disease globally. Airborne pollen is a known environmental trigger, but the impact of different pollen types on pediatric asthma remains unclear. Seasonal and geographic pollen variations, influenced by climate change, may affect asthma patterns. A comprehensive review is needed to clarify these associations and guide prevention strategies.

**Methods:**

Relevant literature on the association between airborne pollen and asthma in children was retrieved from CNKI, Wanfang Data, VIP, CBM, Web of Science, PubMed, Cochrane and Embase at home and abroad from the establishment of the database to March 1, 2025. EndNote X8 and Excel 2021 were used for data management and screening, while Stata 15 was used for statistical analysis.

**Results:**

A total of 9 articles were included in this meta-analysis, from 2007 to 2024, with a total sample size of 87,270 children. The pooled analysis showed that airborne pollen exposure was significantly associated with the risk of childhood asthma (OR = 1.23, 95% CI: 1.13–1.33, *P* < 0.001). Subgroup analysis showed that the combined effect size of tree pollen exposure was OR = 1.56 (95% CI: 0.99–2.12, *P* < 0.001), and the effect size of grass and weed pollen exposure was OR = 1.06 (95% CI: 1.01–1.12, *P* < 0.001). The comprehensive effect size of literature considering air pollutants and climatic factors was OR = 1.35 (95% CI: 1.20–1.50, *P* = 0.034), while literature not considering these factors was OR = 1.08 (95% CI: 1.06–1.10, *P* = 0.156). Age subgroup analysis showed that the effect size for preschool children was OR = 1.31 (95% CI: 0.53–2.09, *P* = 0.156), and for school-age children was OR = 1.52 (95% CI: 1.32–1.73, *P* = 0.298). Sensitivity analysis and Egger's test showed no significant publication bias.

**Conclusion:**

Airborne pollen is a notable risk factor for childhood asthma, and tree pollen exposure may be more dangerous than grass and weed pollen exposure. When atmospheric pollutants and meteorological conditions are considered, the association between airborne pollen and childhood asthma is more pronounced. The evidence is insufficient to support a significant age-related difference between pollen and asthma.

## 1 Introduction

With the changes in people's lifestyles and the acceleration of urbanization, the urban vegetation has steadily increased, and the pollen concentration in the air has also increased accordingly. This may be an important factor contributing to the rising incidence of childhood asthma in recent years (1, 2). According to the World Health Organization (WHO), currently about 10% of children and adolescents worldwide suffer from asthma, affecting over 230 million people in total. Moreover, the disease is characterized by earlier onset and rising prevalence year by year (3, 4). Childhood asthma not only endangers the health of the respiratory system, but also is prone to recurrence and has a prolonged course of disease, seriously affecting their study, life and psychological development, and causing significant social and economic burdens. Airborne pollen is one of the common and important environmental triggers of childhood asthma. It spreads in large quantities in specific seasons and can trigger allergic reactions upon inhalation, inducing airway inflammation and asthma attacks. The intensity and timing of pollen dissemination have obvious geographical and climatic dependencies and are closely related to meteorological factors such as topography, vegetation structure, temperature, humidity and wind speed (5). Due to the significant differences in ecological environment and climatic conditions across regions, the types, concentrations and exposure times of pollen that children in different areas are exposed to are not the same. Coupled with the heterogeneity of individual immune responses, the relationship between pollen exposure and asthma is highly complex and multifactorial (6). Therefore, it is necessary to systematically integrate the existing epidemiological research results through the meta-analysis method, evaluate whether there is a dose-response relationship between airborne pollen exposure and childhood asthma, and clarify its temporal and spatial distribution characteristics in different regions, to inform targeted environmental strategies and improve asthma prevention efforts.

## 2 Materials and methods

### 2.1 Literature search

Cohort studies, case-control studies, cross-sectional studies, time series studies and case-crossover studies on the relationship between airborne pollen and asthma in children were retrieved from CNKI, Wanfang Data, VIP, CBM, Web of Science, PubMed, Cochrane and Embase databases via electronic search. The retrieval time limit was from the establishment of the database to March 1, 2025. The search strategy was (pollen OR aeroallergen OR airborne pollen OR seasonal pollen OR allergic pollen) AND (child OR children OR pediatric OR infant OR adolescent OR preschool) AND (asthma OR wheezing OR bronchial asthma OR allergic asthma OR respiratory allergy). In addition, manually screening the references of included articles, that is, browsing the references of the included research and related review articles to minimize the risk of missing relevant studies.

### 2.2 Inclusion and exclusion criteria

Inclusion criteria: ① Study type: cohort study, case-control study, cross-sectional study, time series and case-crossover study with clear and reproducible data; ② The subjects were children under 18 years old with definite diagnosis of asthma, regardless of gender and race; ③ Exposure was defined as confirmed airborne pollen (such as tree pollen, grass and weed pollen); ④ The outcome measures were to provide extractable effect values, such as OR, RR and 95% CI.

Exclusion criteria ① The original data is incomplete, data duplication, incomplete or unable to extract the effect value; ② Repeated publication, low quality of literature; ③ Review, conference abstracts, commentary articles, etc.; ④ Studies in which comorbidities could affect asthma assessment were excluded.

### 2.3 Literature quality evaluation

The Newcastle-Ottawa Scale (NOS) (7) was used to evaluate the quality of the included cohort studies, case-control studies and case-crossover studies, with a total score of 9 points. The quality assessment was completed independently by two researchers. For studies with inconsistent scores, it was resolved through discussion or arbitration by a third party. The specific contents of the NOS scale include: inclusion criteria compliance (4 items), comparability of research methods (2 items) and data integrity (3 items). The score ≥7 is high quality, 4–6 is medium quality, and <4 is low quality. The cross-sectional study used the Agency for Healthcare Research and Quality scale (AHRQ) (7) for bias risk assessment. The recommended criteria included 11 items, which were answered with “yes”, “no”, “unclear” and “not applicable” respectively. There is currently no quality assessment tool for time series studies. The results of quality assessment showed that most of the included studies were medium and high-quality studies. Sensitivity analysis indicated that excluding low-quality studies did not significantly impact the main findings.

### 2.4 Literature screening and extraction

Two researchers independently used EndNote X8 to screen the literature and extract relevant data, including the following information: ① Author, publication year, study country, study design, and sample size; ② Pollen species and exposure levels; ③ Diagnostic criteria for asthma; ④ Reported effect estimates (OR, RR, and 95% CI); ⑤ Adjusted confounding factors. The screening of titles/abstracts and full texts was performed independently by both reviewers. Any discrepancies in study selection or data extraction were resolved through discussion or by consulting a third reviewer. The extracted data were checked by a third party, and all information was compiled using Excel 2021. Missing data were retrieved by contacting authors or addressed in sensitivity analyses.

### 2.5 Statistical methods

Meta-analysis was performed using Stata 15 software. The effect size was expressed as odds ratio (OR) and its 95% confidence interval (95% CI). Heterogeneity between studies was assessed by *Q* test and *I*^2^ statistic. When *I*^2^ > 50% and *P* < 0.1, significant heterogeneity was considered (8). In the case of significant heterogeneity, a random-effects model (DerSimonian–Laird method) was used to calculate the combined effect; otherwise, a fixed-effects model was used (9). Subgroup analysis was conducted to explore the effects of different pollen species on childhood asthma, the influence of environmental pollution and meteorological conditions on the relationship between airborne pollen and childhood asthma, as well as the differences in responses of children of different age groups to airborne pollen exposure. Sensitivity analysis was performed by excluding studies one by one, and publication bias was assessed by Egger's test and funnel plot.

## 3 Result

### 3.1 Literature retrieval results

**Figure 1:**
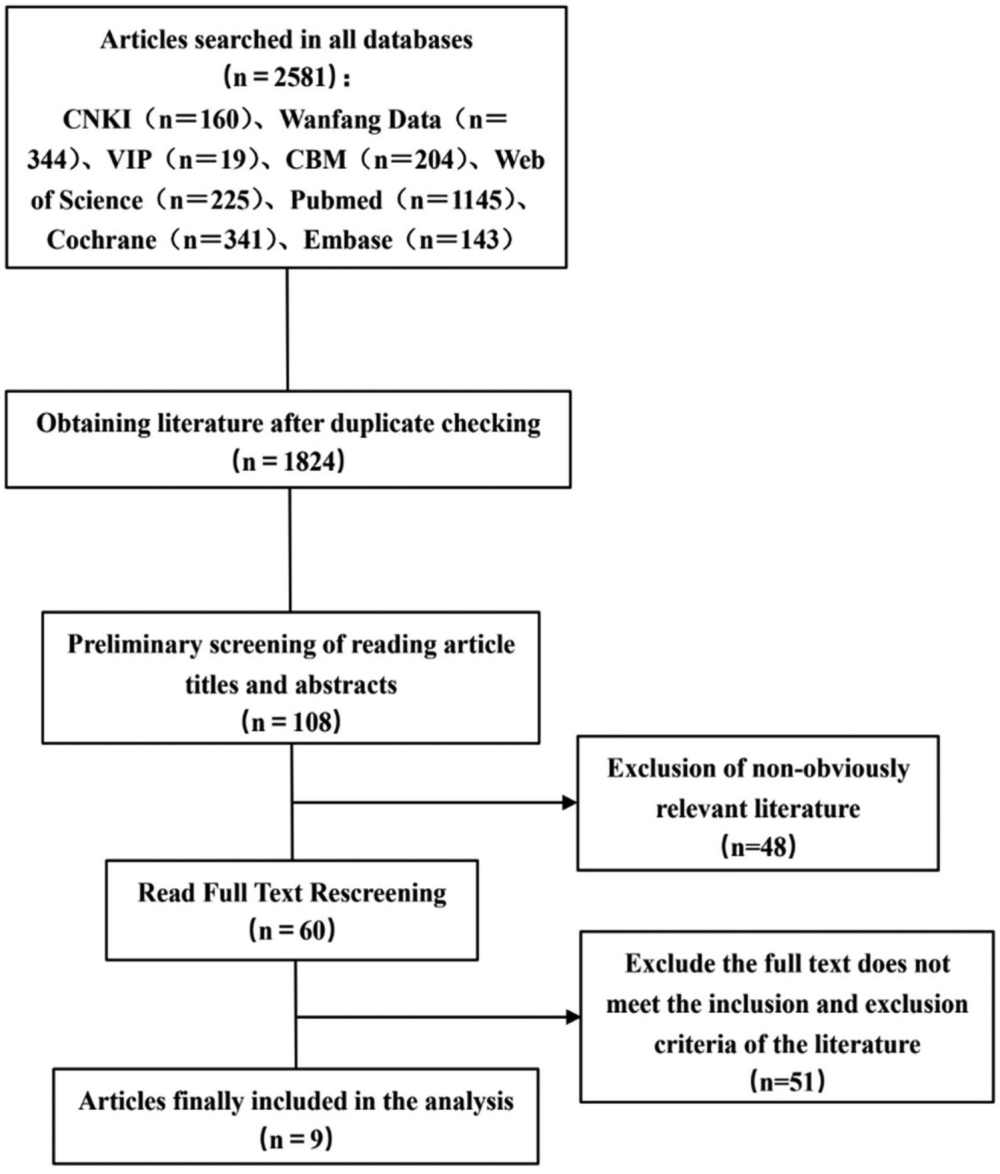
Literature screening flow chart.

A total of 2,581 articles were initially identified through database searches. After removing duplicates, 1,824 articles remained. Screening titles and abstracts yielded 108 articles for full-text review. After applying inclusion and exclusion criteria, 9 articles were finally included. The detailed screening process is illustrated in Figure 1.

### 3.2 Basic information of the literature

A total of 9 articles (all in English) were included in this study, covering the period from 2007 to 2024, with a total sample size of 87,270 children. The specific research types included cohort studies (5 studies), case-crossover studies (2 studies), cross-sectional studies (1 study), and case-control studies (1 study). The research area covered the United States (5 articles), Canada (2 articles), Greece (1 article), and Spain (1 article). The research regions include a total of three climate types: Mediterranean climate, humid subtropical climate and humid continental climate. The main exposure factors were tree pollen (4 articles), grass and weed pollen (5 articles). The specific tree pollens included in the study are olive trees, maple trees, birch trees, oak trees, deciduous trees, evergreen trees, cypress trees, pine trees, mulberry trees, alder trees, elm trees, fraxinus pollen, etc. Grass and weed pollen includes mixed grass, top grass, ragweed, rice, nettle/coarse leaf grass, sage/mugwort, plantago asiatica, salsola, top grass pollen, sikamore pollen, etc. The included studies were all medium and high quality studies. One article scored 9 points, two scored 8 points, five scored 7 points, and one scored 6 points. The basic characteristics of the included studies are summarized in Table 1.

**Table l:** Basic information of included literature.

Author	Year	Study design	*N*	Location	Sort of pollen	Climate type	Exposure indicators	Adjusted covariates	Quality evaluation	Literature resources
Anthracopoulos, M. B. (10)	2007	Cohort study	2,273	Maroussi, Aliartos, Greece	Total pollen (mixed grass, parietaria officinalis, olive trees pollen)	Mediterranean climate	Questionnaires	No	7	《Ann Allergy Asthma Immunol》
De Roos, Anneclaire J. (11)	2024	Case-crossover study	2022	Philadelphia, Pennsylvania, USA	Early-season tree pollen (maple trees, birch trees, oak trees pollen), early-season grass and weed pollen (ragweed, rice pollen)	Humid subtropical climate	Acute attack frequency of asthma	Respiratory virus infection, temperature, humidity, mold, O_3_, particulate matter	8	《The journal of allergy and clinical immunology. Global》
DellaValle, C. T. (12)	2012	Cohort study	430	Connecticut, South-central Massachusetts and New York, USA	Total pollen, tree pollen, grass and weed pollen (ragweed pollen)	Humid continental climate	Severity of asthma	Maximum daily temperature, maximum 8-h average O_3_, PM_2.5_, season and antibiotic use	6	《Epidemiology》
Duquesne, Louise (13)	2023	Cohort study	30,816	Montreal, Canada	Total pollen (deciduous trees, evergreen trees pollen)	Humid continental climate	Incidence of asthma	Urban total vegetation, tree types and grasses	9	《Environmental Epidemiology》
Gleason, J. A. (14)	2014	Case-crossover study	21,854	New jersey, USA	Tree pollen, grass and weed pollen (ragweed pollen)	Humid subtropical climate	Emergency treatment rate	PM_2.5_, average temperature and average humidity, school hours and holiday indicators	7	《Environ Res》
Harley, K. G. (15)	2009	Cohort study	514	Salinas Valley, California, USA	Tree pollen (cypress 32%, oak 11%, pine 7%, mulberry 4%, alder 4%, elm 3%), grass and weed pollen (nettle/rough leaf grass 4%, sage/mugwort 2%, plantago asiatica 2%, etc.)	Mediterranean climate	Early wheezing	Environmental PM_2.5_ levels and lower respiratory tract infection	7	《Thorax》
Segura, N. (16)	2016	Cross section study	468	Zaragoza, Spain	Grass pollen (salsola, parietaria pollen)	Mediterranean climate	ISAAC Questionnaire	Family history of asthma and environmental risks (exposure to tobacco smoke, livestock; history of rhinitis, eczema or lower respiratory tract disease)	7	《Allergol Immunopathol (Madr)》
Stanescu, C. (17)	2024	Cohort study	28,543	Toronto, Canada	Tree pollen, grass and weed pollen (no specific pollen types were mentioned)	Humid continental climate	Ontario ASTHMA Queue Database	The season of pollen	8	《Eur Respir J》
Weinberger (18)	2015	Case control study	350	Almonk, New York, USA	Tree pollen (maple trees, birch trees, fraxinus pollen), grass and weed pollen (sikamore pollen)	Humid continental climate	Emergency treatment rate	Temperature and carbon dioxide concentration, O_3_ and PM_2.5_, canopy coverage, black carbon, median household income and percentage of non-Hispanic blacks.	7	Masteral dissertation

### 3.3 Meta-analysis results of the relationship between airborne pollen and childhood asthma

**Figure 2:**
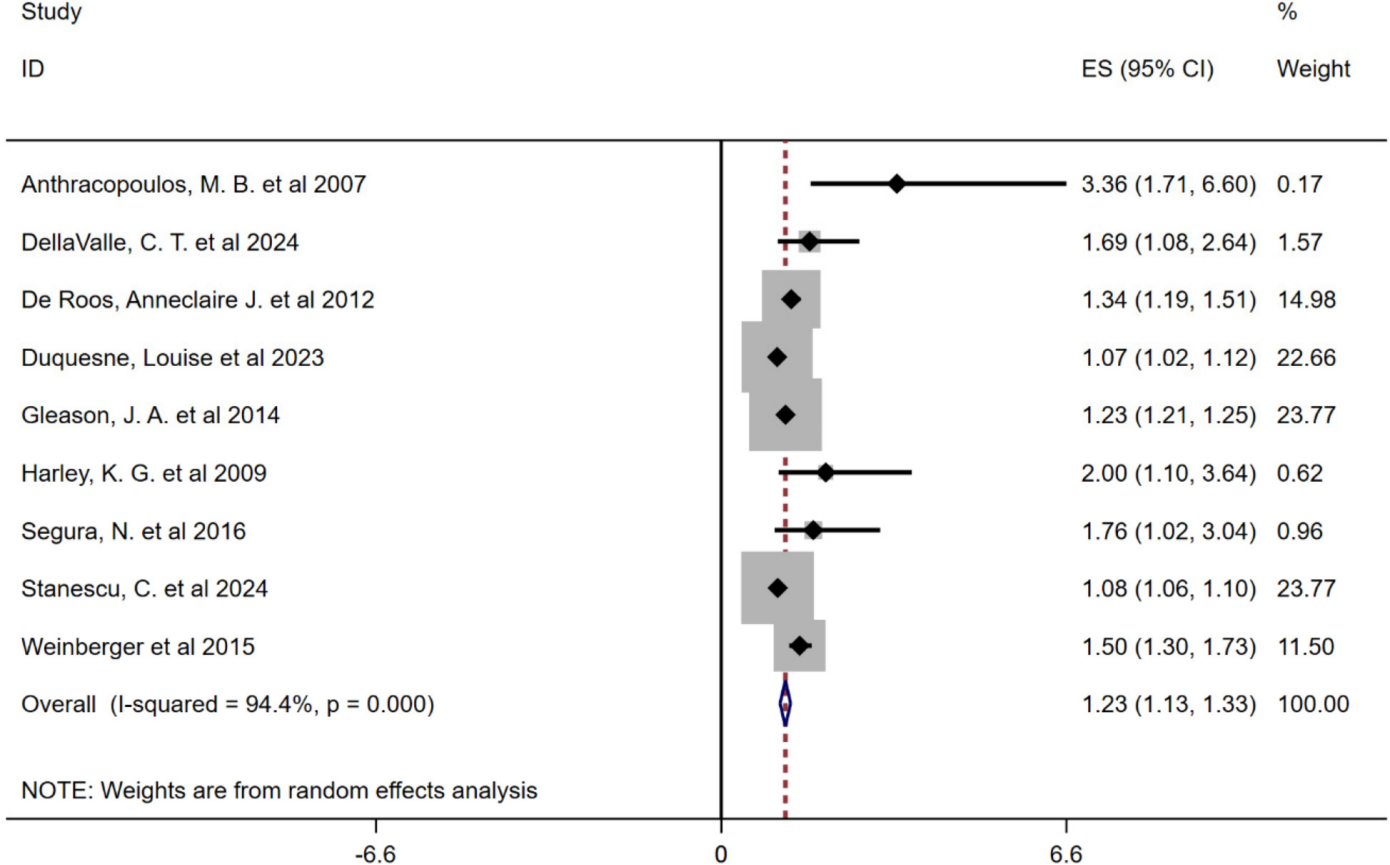
Meta-analysis of the relationship between airborne pollen and asthma in children forest plot.

The heterogeneity test revealed significant variability among studies (*I*^2^ = 94.4%, *P* < 0.001), prompting the use of a random-effects model. The combined results indicated a significant association between airborne pollen exposure and increased risk of childhood asthma (OR = 1.23, 95% CI: 1.13–1.33, *P* < 0.001), as shown in Figure 2.

**Figure 3:**
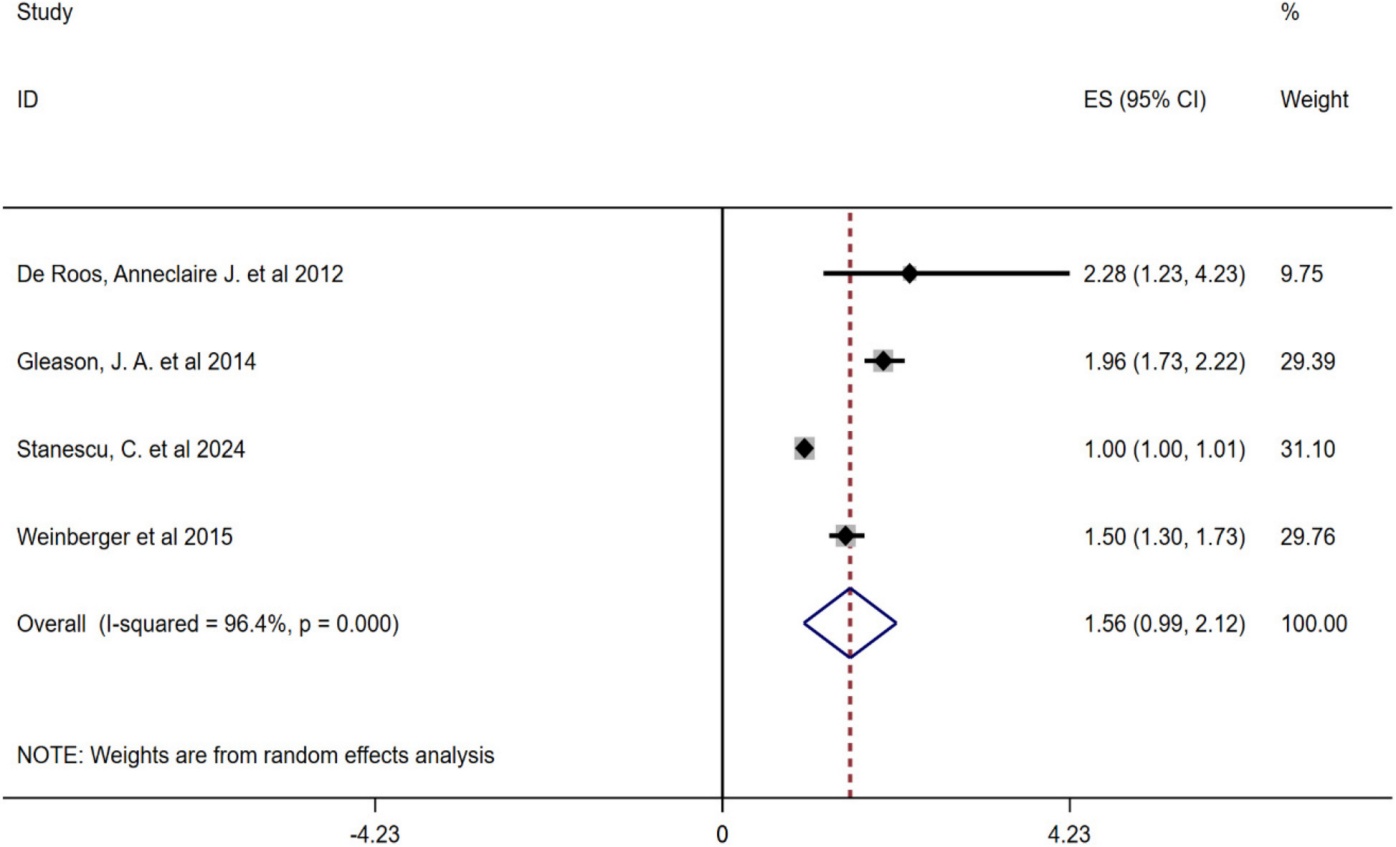
Subgroup analysis of the relationship between tree pollen and childhood asthma forest plot.

**Figure 4:**
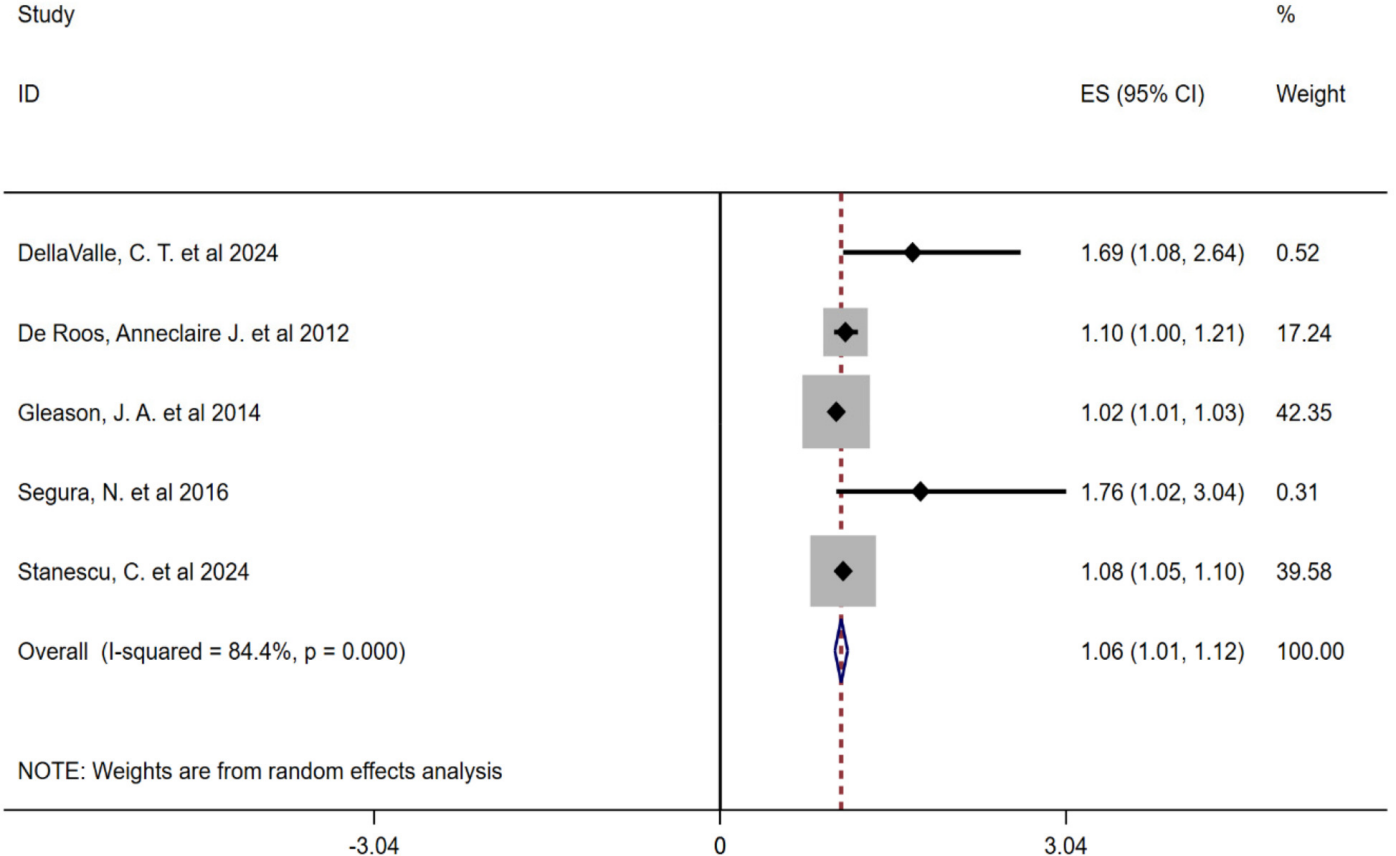
Subgroup analysis forest plot of the association between grass and weed pollen and childhood asthma.

Due to the high heterogeneity between the included studies, this study conducted a subgroup analysis of pollen types. Heterogeneity analysis showed that there was a high degree of heterogeneity between the studies, so a random-effects model was also used to combine the effect sizes. The results showed that the combined effect size of tree pollen exposure was OR = 1.56 (95% CI: 0.99–2.12, *P* < 0.001), and that of grass and weed pollen exposure was OR = 1.06 (95% CI: 1.01–1.12, *P* < 0.001), see Figures 3, 4.

**Figure 5:**
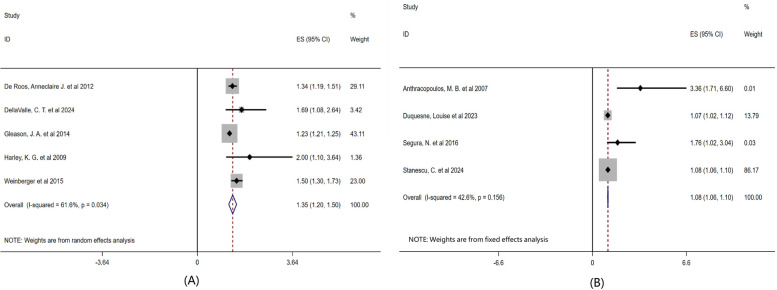
Subgroup analysis of the effects of air pollutants and climatic factors on childhood asthma forest plot. Note: **(A)** Taking into account the influence of air pollutants and climatic factors on pollen concentration; **(B)** The influence of atmospheric pollutants and climatic factors on pollen concentration was not taken into account.

Secondly, to further explore the differences in the relationship between airborne pollen and childhood asthma under different levels of environmental exposure, this study conducted a subgroup analysis based on atmospheric pollutants and meteorological factors, as shown in Figure 5. The results showed that the comprehensive effect size of the literature considering air pollutants and climate factors was OR = 1.35 (95% CI: 1.20–1.50, *P* = 0.034), while that of the literature not considering air pollutants and climate factors was OR = 1.08 (95% CI: 1.06–1.10, *P* = 0.156).

**Figure 6:**
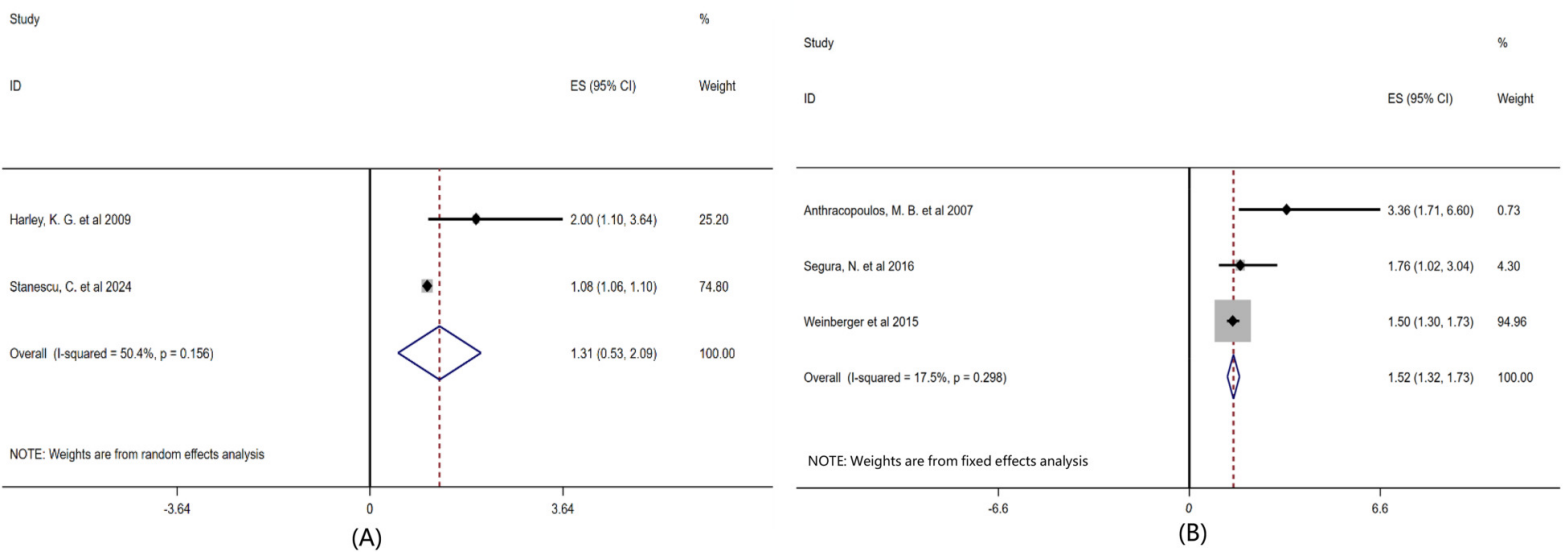
Subgroup analysis of the influence of age factor on childhood asthma forest plot. Note: Grouped according to the age of the existing included literature: **(A)** Preschool children < 6 years old, **(B)** School-age children 6-14 years old.

To explore the differences in responses of children of different age groups to airborne pollen exposure, we conducted subgroup analyses based on the average age or age distribution of the children, as shown in Figure 6. The children patients were divided into two groups: preschool children (<6 years old) and school-age children (6–14 years old). Age subgroup analysis showed that the comprehensive effect size of children under 6 years old was OR = 1.31 (95% CI: 0.53–2.09, *P* = 0.156), and that of children aged 6–14 years old was OR = 1.52 (95% CI: 1.32–1.73, *P* = 0.298).

### 3.4 Publication bias and sensitivity analysis

**Figure 7:**
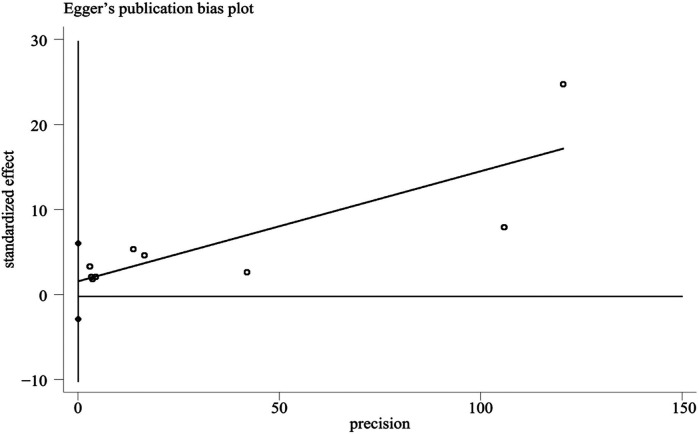
Funnel plot of included literature bias analysis.

The funnel plot was used to evaluate publication bias. The Egger's test was performed on the literature included in this study. The results showed that the distribution of the study was roughly symmetrical, and there was no obvious small sample effect. The Egger's test results were *t* = 0.94, *P* = 0.378, indicating that no significant publication bias was found in this study (Figure 7).

**Figure 8:**
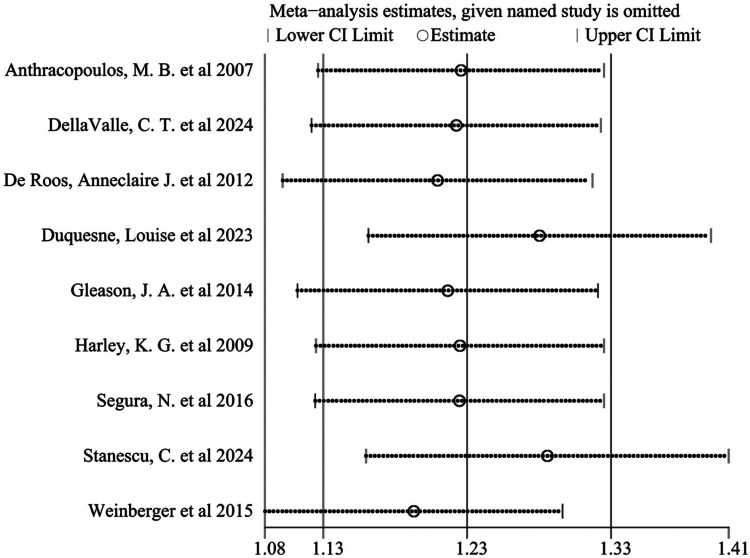
Sensitivity analysis chart of included literature.

Sensitivity analysis was performed by sequentially excluding each study. The results indicated that no single study substantially influenced the overall effect, demonstrating the robustness of the findings (Figure 8). Furthermore, effect estimates from both fixed-effect (OR = 1.15, 95% CI: 1.14–1.17) and random-effect models (OR = 1.23, 95% CI: 1.13–1.33) were consistent, further supporting result stability.

## 4 Discussion

In this study, we systematically integrated 9 studies (including 5 cohort studies, 2 case-crossover studies, 1 cross-sectional study, 1 case-control study), a total of 87,270 children's data, covering the United States, Canada, Greece, Spain 4 countries. Our analysis revealed that airborne pollen exposure was significantly associated with the risk of childhood asthma (OR = 1.23, 95% CI: 1.13–1.33, *P* < 0.001), and subgroup analysis indicated that different pollen species had different effects on childhood asthma. The combined effect size of tree pollen exposure was OR = 1.56 (95% CI: 0.99–2.12, *P* < 0.001), and the effect size of grass and weed pollen exposure was OR = 1.06 (95% CI: 1.01–1.12, *P* < 0.001). These results suggest that tree pollen exposure may have a more pronounced impact on childhood asthma.

There are significant differences in the effects of tree pollen and grass pollen on childhood asthma, which are mainly reflected in the differences in their pollen types and transmission times. A specific analysis of the regional data of the four countries involved in the above-mentioned literature reveals that tree pollen usually peaks in spring each year. For instance, in the United States, Spain and Greece, it is concentrated from March to May, while in Canada, it is from April to June. Its main sources include birch, maple, oak, olive, cypress and pine, etc. Grass pollen, on the other hand, is commonly found from August to September each year, with ragweed as a representative, showing a relatively uniform seasonal peak. The specific time varies depending on the regional climate. This seasonal characteristic indicates that effective preventive measures rely on regionalized pollen calendars and real-time monitoring systems to accurately warn of the high-incidence periods of pollen. For highly sensitive children, public health strategies should include timely release of pollen forecasts, promotion of avoidance behaviors during high-risk periods, optimization of the timing of drug interventions (such as antihistamines or corticosteroids), and limiting outdoor activities or using high-efficiency particulate air filters indoors during peak pollen periods. In addition, allergen-specific immunotherapy can be customized for the dominant pollen types in each region. By scientifically distinguishing pollen types, paying attention to the high incidence period of pollen, taking targeted protective measures, and combining pollen monitoring and individualized management, the risk of asthma attacks in children can be effectively reduced.

Furthermore, the comprehensive effect size of the literature that included air pollutants and climatic factors was OR = 1.35 (95% CI: 1.20–1.50, *P* = 0.034), while that of the literature that did not include air pollutants and climatic factors was OR = 1.08 (95% CI: 1.06–1.10, *P* = 0.156). Therefore, under the premise of considering atmospheric pollutants and meteorological factors, it was found that the association between pollen exposure and the risk of childhood asthma was stronger, indicating that the impact of pollen on childhood asthma does not occur in isolation but is magnified under a certain climate and pollution background. The children patients were divided into two groups: preschool children and school-age children. Age subgroup analysis showed that the comprehensive effect size of children under 6 years old was OR = 1.31 (95% CI: 0.53–2.09, *P* = 0.156), and that of children aged 6–14 years old was OR = 1.52 (95% CI: 1.32–1.73, *P* = 0.298), suggesting that school-age children may be more sensitive to pollen exposure, but the difference is not statistically significant. Currently, the evidence is insufficient to support that age has a significant regulatory effect on the relationship between pollen and asthma.

At present, the specific biological mechanism of the effect of airborne pollen on childhood asthma is still unclear. This may be attributed to protease antigens present in pollen particles, which trigger IgE-mediated hypersensitivity upon inhalation, resulting in airway spasms, mucosal edema, and airway obstruction (19, 20). It may also be due to the protease in pollen grains destroying the lung epithelial barrier, resulting in increased transepithelial permeability and allergen exposure, resulting in sensitization to various allergens (21). Studies have found that pollen allergens trigger type 2-driven immune responses by interacting with various molecular factors and lipids, causing allergic diseases such as allergic rhinitis and asthma (22). Animal models have also provided experimental support for these mechanisms. Although these models cannot fully replicate all the characteristics of human asthma, they are of great value in revealing the disease mechanism and screening potential treatment methods (23). For example, type 2 helper T cells (Th2) play a core role in airway inflammation and airway hyperresponsiveness (AHR), and dendritic cells, T cell receptors, major histocompatibility complexes (MHC), and costimulatory molecules are also crucial (24, 25). Such experimental evidence strengthens the plausibility of our meta-analysis results, suggesting a causal role of pollen exposure in asthma. While our meta-analysis provides epidemiological evidence for the association between airborne pollen and pediatric asthma, it lacks direct mechanistic data. Future studies should integrate epidemiological surveillance with experimental models to elucidate the molecular and immunological pathways involved.

The results of this study are consistent with the consensus of previous literature, which further verifies that airborne pollen exposure will increase the risk of asthma attacks in children, but the research on pollen species is different. For example, a systematic review and meta-analysis by Erbas et al. (26) found that outdoor pollen concentration was positively correlated with the performance of children and adolescents in emergency department visits, and exposure to surrounding grass pollen was an important trigger for asthma attacks in children in emergency department visits. A systematic review and meta-analysis by Shrestha et al. (27) also found a possible association between grass and birch pollen and childhood asthma hospitalization. An increase of 10 grass pollen grains per m^3^ was associated with a 3% increase in childhood asthma hospitalization and an increase of 10 birch pollen grains per m^3^ was significantly associated with an average percentage change in childhood asthma hospitalization. But Yu et al. (28) found that exposure to trees may reduce the risk of childhood asthma, while exposure to grassland may increase the risk. Damialis et al. (29) found that low exposure during the pollen peak season can effectively reduce allergic symptoms and immune responses. Therefore, avoiding exposure to allergens through environmental control is one of the effective means to prevent asthma attacks in children.

Although this study fully explored the relationship between airborne pollen and childhood asthma, there are some limitations. First of all, there are differences in the selection of exposure assessment methods in the included studies. The diversity of exposure assessment methods also increases the complexity of result integration. Secondly, due to the regional and seasonal differences of allergenic pollen (30), the high exposure standards in different regions are not uniform, making it difficult to determine the critical exposure threshold of asthma attacks, thus affecting the formulation of preventive measures. Finally, most of the current included studies are small sample size and data from a single institution. In the future, it is necessary to further enhance the reliability and universality of the conclusions by expanding the sample size and implementing multi-center collaborative research.

## 5 Conclusion

Airborne pollen is a significant risk factor for childhood asthma, with exposure to tree pollen potentially posing a greater threat than grass and weed pollen. When atmospheric pollutants and meteorological conditions are taken into account, the association between airborne pollen exposure and childhood asthma becomes more pronounced. Currently, there is insufficient evidence to support a significant age-related difference in the relationship between pollen exposure and asthma risk. Therefore, the airborne pollen exposure index may serve as a valuable intervention target for precise environmental control, representing a critical strategy in the prevention and management of childhood asthma. Concurrently, dynamic monitoring of asthma control in children, coupled with health education on effective management strategies, should be implemented to facilitate early intervention, ensure adequate protection, and optimize treatment outcomes, thereby minimizing adverse health impacts on children and alleviating the burden on public health systems.

## Data Availability

The raw data supporting the conclusions of this article will be made available by the authors, without undue reservation.

## References

[1] Parmes E Pesce G Sabel CE Baldacci S Bono R Brescianini S Influence of residential land cover on childhood allergic and respiratory symptoms and diseases: evidence from 9 European cohorts. Environ Res. (2019) 179: 108953. 10.1016/j.envres.2019.108953 31818476

[2] Lovasi GS Neil-Dunne O Lu JP Sheehan JW Perzanowski D Macfaden MS Urban tree canopy and asthma, wheeze, rhinitis, and allergic sensitization to tree pollen in a New York City birth cohort. Environ Health Perspect. (2013) 121(4):494–500. 10.1289/ehp.1205513 23322788 PMC3620770

[3] Kolosova N Denisova V Shatalina S. Management of childhood asthma: what is new? Med Sovet. (2024) 19:52–7. 10.21518/ms2024-416

[4] Poole J. Asthma is a major noncommunicable disease affecting over 230 million people worldwide and represents the most common chronic disease among children. Int Immunopharmacol. (2014) 23(1):315. 10.1016/j.intimp.2014.09.013 25305594

[5] Frisk C Brobakk T Rizzi J Ramfjord H. Influence of spatiotemporal and meteorological variation on Norwegian atmospheric pollen seasonality. Agric Meteorol. (2024) 353:110059. 10.1016/j.agrformet.2024.110059

[6] Simunovic M Boyle J Erbas B Baker P Davies JM. Airborne grass pollen and thunderstorms influence emergency department asthma presentations in a subtropical climate. Environ Res. (2023) 236:116754. 10.1016/j.envres.2023.116754 37500047

[7] Zeng X Liu H Chen X Leng W. Meta-analysis series IV: quality assessment tools for observational studies. Chin J Evid-Based Cardiovasc Med. (2012) 4(4):297–9.

[8] Higgins JP Thompson SG Deeks JJ Altman DG. Measuring inconsistency in meta-analyses. Br Med J. (2003) 327(7414):557–60. 10.1136/bmj.327.7414.557 12958120 PMC192859

[9] Myles P Kasza J Turner T. Credibility of subgroup findings in clinical trials and meta-analyses. Br J Anaesth. (2021) 127(1):11–4. 10.1016/j.bja.2021.04.007 33992396

[10] Anthracopoulos MB Mantzouranis E Paliatsos AG Tzavelas G Lagona E Nicolaidou P Different effects of sensitization to mites and pollens on asthma symptoms and spirometric indices in children: a population-based cohort study. Ann Allergy Asthma Immunol. (2007) 99(2):122–9. 10.1016/S1081-1206(10)60635-7 17718099

[11] De Roos AJ Senter JP Schinasi LH Huang W Moore K Maltenfort M Outdoor aeroallergen impacts on asthma exacerbation among sensitized and nonsensitized Philadelphia children. J Allergy Clin Immunol Glob. (2024) 3(3):100248. 10.1016/j.jacig.2024.100248 38645670 PMC11024998

[12] DellaValle CT Triche EW Leaderer BP Bell ML. Effects of ambient pollen concentrations on frequency and severity of asthma symptoms among asthmatic children. Epidemiology. (2012) 23(1):55–63. 10.1097/EDE.0b013e31823b66b8 22082997 PMC3246281

[13] Duquesne L Anassour Laouan Sidi E Plante C Liu Y Zhao N Lavigne É The influence of urban trees and total vegetation on asthma development in children. Environ Epidemiol. (2023) 7(6):e280. 10.1097/EE9.0000000000000280 38912389 PMC11189683

[14] Gleason JA Bielory L Fagliano JA. Associations between ozone, PM2.5, and four pollen types on emergency department pediatric asthma events during the warm season in New Jersey: a case-crossover study. Environ Res. (2014) 132:421–9. 10.1016/j.envres.2014.03.035 24858282

[15] Harley KG Macher JM Lipsett M Duramad P Holland NT Prager SS Fungi and pollen exposure in the first months of life and risk of early childhood wheezing. Thorax. (2009) 64(4):353–8. 10.1136/thx.2007.090241 19240083 PMC3882001

[16] Segura N Fraj J Cubero JL Sobrevía MT Lezaun A Ferrer L Mould and grass pollen allergy as risk factors for childhood asthma in Zaragoza, Spain. Allergol Immunopathol (Madr). (2016) 44(5):455–60. 10.1016/j.aller.2016.02.003 27255475

[17] Stanescu C Talarico R Weichenthal S Villeneuve PJ Smargiassi A Stieb DM Early life exposure to pollens and increased risks of childhood asthma: a prospective cohort study in Ontario children. Eur Respir J. (2024) 63(4):2301568. 10.1183/13993003.01568-2023 38636971 PMC11025571

[18] Weinberger KR. Spatial and temporal distribution of tree pollen in New York city: linking aeroallergen measurements to health (Dissertation). ProQuest Dissertations & Theses Global, New York (2015). Available online at: https://www.proquest.com/dissertations-theses/spatial-temporal-distribution-tree-pollen-new/docview/1719270417/se-2

[19] Pointner L Bethanis A Thaler M Traidl-Hoffmann C Gilles S Ferreira F Initiating pollen sensitization—complex source, complex mechanisms. Clin Transl Allergy. (2020) 10:36. 10.1186/s13601-020-00341-y 32884636 PMC7461309

[20] León B. Understanding the development of Th2 cell-driven allergic airway disease in early life. Front Allergy. (2023) 3:1080153. 10.3389/falgy.2022.1080153 36704753 PMC9872036

[21] Gaspar R de Matos MR Cortes L Nunes-Correia I Todo-Bom A Pires E Pollen proteases play multiple roles in allergic disorders. Int J Mol Sci. (2020) 21:3578. 10.3390/ijms21103578 32438574 PMC7278992

[22] Guryanova SV Finkina EI Melnikova DN Bogdanov IV Bohle B Ovchinnikova TV. How do pollen allergens sensitize? Front Mol Biosci. (2022) 9:900533. 10.3389/fmolb.2022.900533 35782860 PMC9245541

[23] Shin Y Takeda K Gelfand EW. Understanding asthma using animal models. Allergy Asthma Immunol Res. (2009) 1(1):10–8. 10.4168/aair.2009.1.1.10 20224665 PMC2831565

[24] Leong K Huston D. Understanding the pathogenesis of allergic asthma using mouse models. Ann Allergy Asthma Immunol. (2001) 87(2):96–109; quiz 110. 10.1016/S1081-1206(10)62201-6 11527255

[25] Takeda K Dakhama A Gelfand EW. Allergic asthma: what have we learned from the mouse model? Allergol Int. (2005) 54(3):263–71. 10.2332/allergolint.54.263

[26] Erbas B Jazayeri M Lambert KA Katelaris CH Prendergast LA Tham R Outdoor pollen is a trigger of child and adolescent asthma emergency department presentations: a systematic review and meta-analysis. Allergy. (2018) 73(8):1632–41. 10.1111/all.13407 29331087

[27] Shrestha SK Lambert KA Erbas B. Ambient pollen concentrations and asthma hospitalization in children and adolescents: a systematic review and meta-analysis. J Asthma. (2020) 58(10):1155–68. 10.1080/02770903.2020.1771726 32419541

[28] Yu H Zhou Y Wang R Qian Z Knibbs LD Jalaludin B Associations between trees and grass presence with childhood asthma prevalence using deep learning image segmentation and a novel green view index. Environ Pollut. (2021) 286:117582. 10.1016/j.envpol.2021.117582 34438500

[29] Damialis A Häring F Gökkaya M Rauer D Reiger M Bezold S Human exposure to airborne pollen and relationships with symptoms and immune responses: indoors versus outdoors, circadian patterns and meteorological effects in alpine and urban environments. Sci Total Environ. (2019) 653:190–9. 10.1016/j.scitotenv.2018.10.366 30408667

[30] Xiao L Wang C Song T Ma Y Liu B Yue L Research progress on pollen exposure characteristics and sensitization risk assessment in urban green space. China Urban Green. (2022) 20(6):159–67.

